# Supplementary material for: Treatment of Critical Size Femoral Bone Defects with Biomimetic Hybrid Scaffolds of 3D Plotted Calcium Phosphate Cement and Mineralized Collagen Matrix
Source: Int J Mol Sci. 2022 Mar 21;23(6):3400. doi: 10.3390/ijms23063400 (PMC8949113; doi:10.3390/ijms23063400)
Supplement: Supplementary file 1 [file ijms-23-03400-s001.zip › ijms-1641142-supplementary.pdf]

International Journal of *Molecular Sciences*

MDPI

supplementary materials

Treatment of critical size femoral bone defects with biomimetic hybrid scaffolds of 3D plotted calcium phosphate cement and mineralized collagen matrix

Anna Carla Culla[1,2,3], Corina Vater[1,2], Xinggui Tian[1,2*], Julia Bolte[1,2], Tilman Ahlfeld[2], Henriette Bretschneider[1,2], Alexander Pape [1,2], Stuart B. Goodman[4], Michael Gelinsky[2] and Stefan Zwingenberger[1,2]

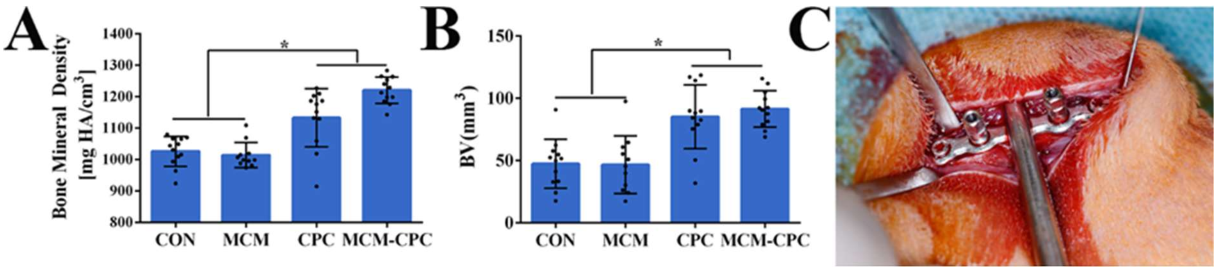

Figure S1. Evaluation of bone defect healing 8 weeks after surgery. **(A):** bone mineral density (BMD) and **(B)**: bone volume (BV) as measured by μCT. **(C):** Rat femoral defect model showing defect area and fixation device (mean ± SD, *$p<0.05$).
